# Extreme Short Bowel Syndrome: A Case of Resilience and Recovery From Acute Intestinal Failure

**DOI:** 10.7759/cureus.85370

**Published:** 2025-06-04

**Authors:** Shariful Islam, Richard Harkissoon, Anthony Maughn, Patrick Harnarayan, Vijay Naraynsingh

**Affiliations:** 1 Department of General Surgery/Oncoplastic Breast Surgery, San Fernando General Hospital, San Fernando, TTO; 2 Department of Clinical Surgical Sciences, University of the West Indies, St. Augustine, TTO; 3 Department of General Surgery, San Fernando General Hospital, San Fernando, TTO; 4 Department of Vascular Surgery, San Fernando General Hospital, San Fernando, TTO; 5 Department of Vascular Surgery, Medical Associates Hospital, St. Joseph, TTO

**Keywords:** acute mesenteric ischemia (ami), extreme short bowel syndrome, intestinal adaptation, intestinal failure, short bowel syndrome (sbs), unexpected outcome

## Abstract

Short bowel syndrome (SBS) is a well-recognized surgical condition that often requires prolonged resuscitation and coordinated multidisciplinary management. Severe cases of SBS are typically associated with high mortality rates. We present the case of a 68-year-old woman diagnosed with ischemic necrosis involving nearly the entire small intestine, extending from 15 cm beyond the duodenojejunal flexure to the proximal transverse colon. Surgical resection of the affected small and large bowel segments was performed, followed by damage control laparotomy. After stabilization in the ICU, a primary anastomosis of the remaining bowel was completed. With intensive resuscitation, multidisciplinary care, and nutritional support, the patient has remained clinically stable for over two years with minimal outpatient intervention following hospital discharge. This case underscores the potential for recovery even in the most severe presentations of SBS and highlights the importance of persistent, collaborative care.

## Introduction

Short bowel syndrome (SBS) is well documented in the literature and is clinically recognized by the presence of malabsorption, diarrhea, steatorrhea, fluid and electrolyte imbalances, and malnutrition resulting from intestinal failure of various etiologies [1]. However, there is no definitive guideline in the English literature regarding the minimum length of bowel required for survival or compatibility with life.

It has been suggested that nutritional autonomy may be achieved with 110-150 cm of small bowel or 50-70 cm of small bowel in continuity with the colon [2]. The average time to achieve nutritional autonomy is approximately 19 months, with a range of three to 126 months [3]. Most causes of SBS and the resulting intestinal failure can be broadly classified into anatomic or functional categories [4].

Globally, SBS affects approximately 3-4% of the population, with 15% of these cases occurring in adults. Among these, 70% of patients survive beyond one year after the etiological event [5].

The current case highlights an elderly, active female with a history of hypertension, cerebrovascular accident (CVA), and atrial fibrillation who presented with extensive bowel necrosis likely due to a thromboembolic event. The patient underwent prolonged surgical and medical management and met all criteria for safe discharge to home and outpatient care - these included successful resuscitation in the ICU, no further extension of ischemia, a successful primary anastomosis during the second-look laparotomy, weaning from ventilatory and inotropic support, stepwise transition from total parenteral nutrition (TPN) to oral feeding, and stable transfer to the surgical ward.

This case illustrates resilience in surgical care within a resource-limited setting and demonstrates the potential for favorable clinical and operative outcomes despite significant surgical and pathological challenges.

## Case presentation

A 68-year-old active female, nonsmoker, hypertensive, with a history of atrial fibrillation and CVA in 2015, presented to the ED with a two-day history of sudden onset “lancing” abdominal pain. The pain was accompanied by multiple episodes of nonbilious, nonbloody vomiting during the same period. She reported no bowel movements throughout the duration of symptoms, while urinary function remained normal. Her history revealed that systemic anticoagulation therapy had been held for one month in anticipation of a dental procedure.

On examination, the patient was hypotensive, with a heart rate of 98 beats per minute, a respiratory rate of 20 breaths per minute, and normal oxygen saturation. Abdominal examination showed diffuse tenderness, especially in the lower abdomen, with mild guarding but no distension. Bowel sounds were absent, and digital rectal examination was unremarkable.

An echocardiogram revealed no cardiac thrombus or cardiomyopathy, with an ejection fraction greater than 60%, while the ECG showed sinus rhythm with premature atrial contractions. Laboratory workup revealed an elevated white blood cell count, normal hemoglobin and platelet counts, normal serum amylase and electrolytes, and a mildly elevated serum creatinine level. Venous blood gas analysis demonstrated partially compensated respiratory alkalosis (Table 1).

**Table 1:** Summary of laboratory investigation results Bold values indicate significant findings.

Category	Test	Result	Reference range
Hematology	Complete blood count		
	White blood cell	19.93	4.10-11.20 × 10³/μL
	Hemoglobin	15	11.7-15.5 g/dL
	Platelet	262	150-350 × 10³/μL
Biochemistry	Sodium	145	135-148 mmol/L
	Potassium	4	3.5-5.1 mmol/L
	Chloride	105.5	97-110 mmol/L
	Blood urea nitrogen	18	7.8-20.2 mg/dL
	Creatinine	1.5	0.5-1.0 mg/dL
	Troponin	0.06	0-0.04 ng/mL
	Amylase	90	40-140 U/L
Venous blood gas	pH	7.502	7.35-7.45
	PCO₂	29.8	35-59 mmHg
	PO₂	109	25-70 mmHg
	HCO₃⁻	23.4	22-30 mmol/L
	Base excess	0	-2 to +2 mmol/L
	Lactate	3.48	0.5-2.0 mmol/L

**Figure 1:**
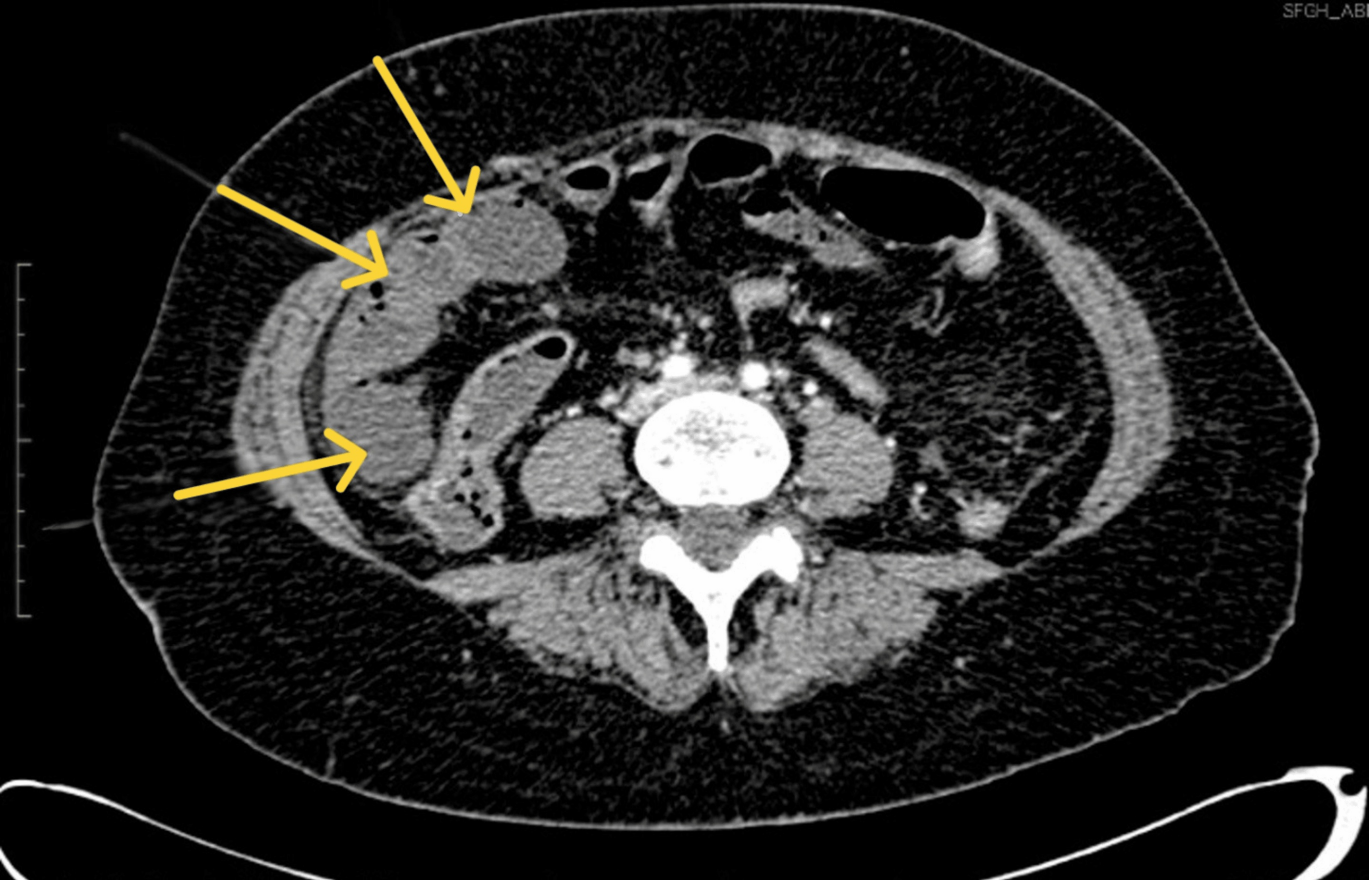
Cross-sectional CT scan of the abdomen and pelvis demonstrating nonenhancing small bowel segments (yellow arrows)

**Figure 2:**
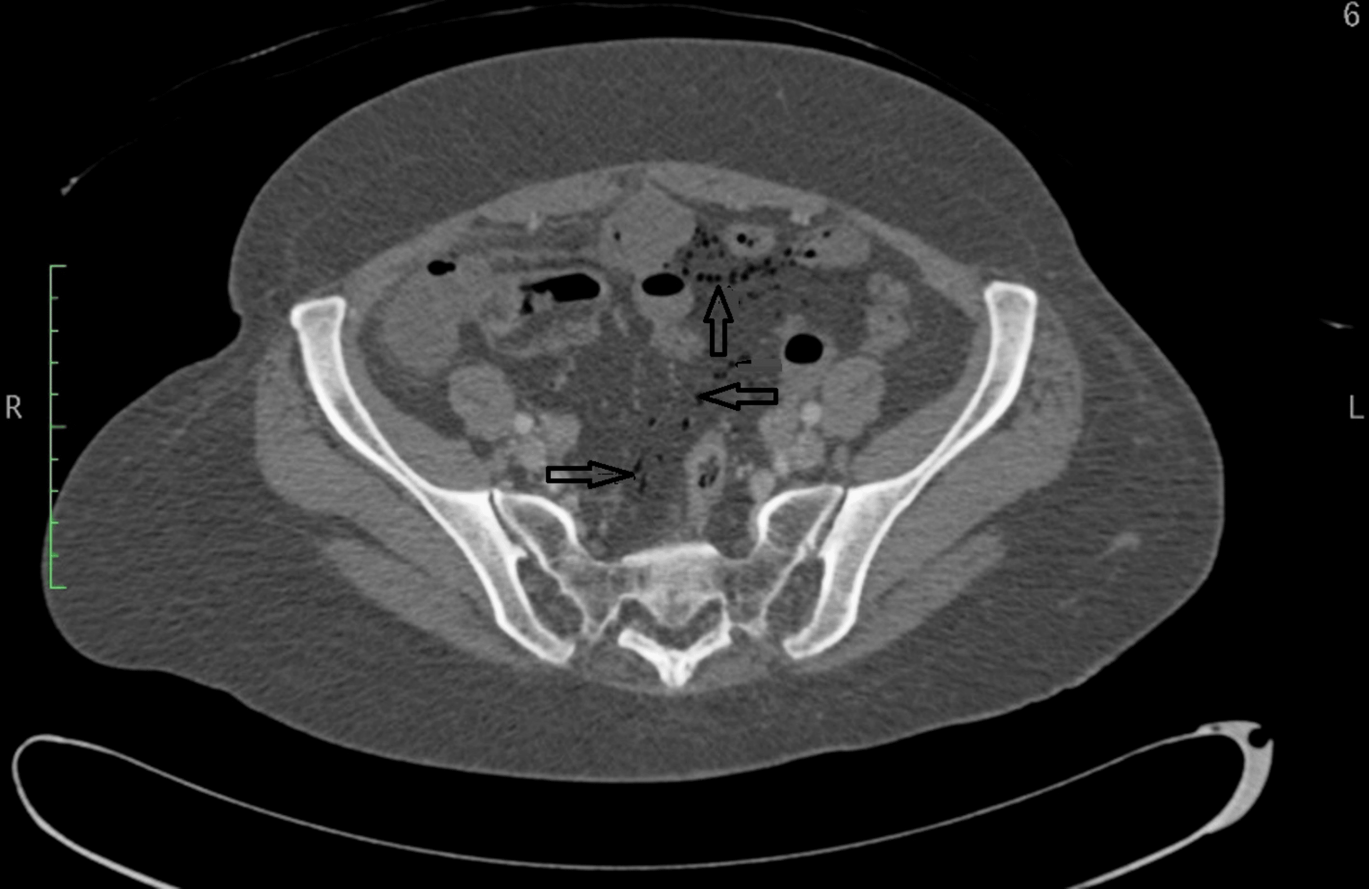
Cross-sectional CT scan of the abdomen and pelvis showing diffuse pneumatosis intestinalis (black arrows)

**Figure 3:**
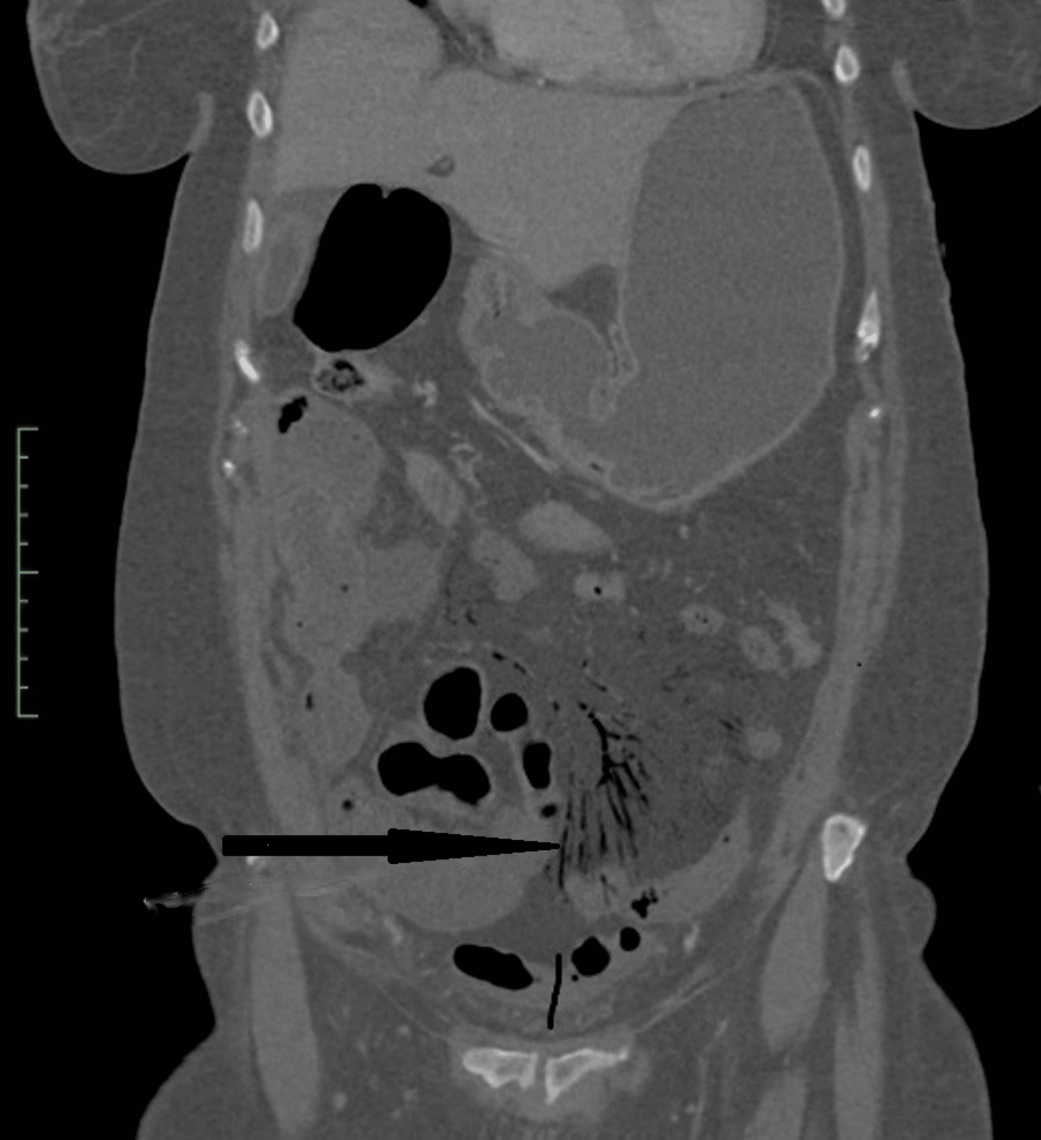
Coronal CT scan view of the abdomen and pelvis revealing mesenteric intravascular gas (black arrow)

**Figure 4:**
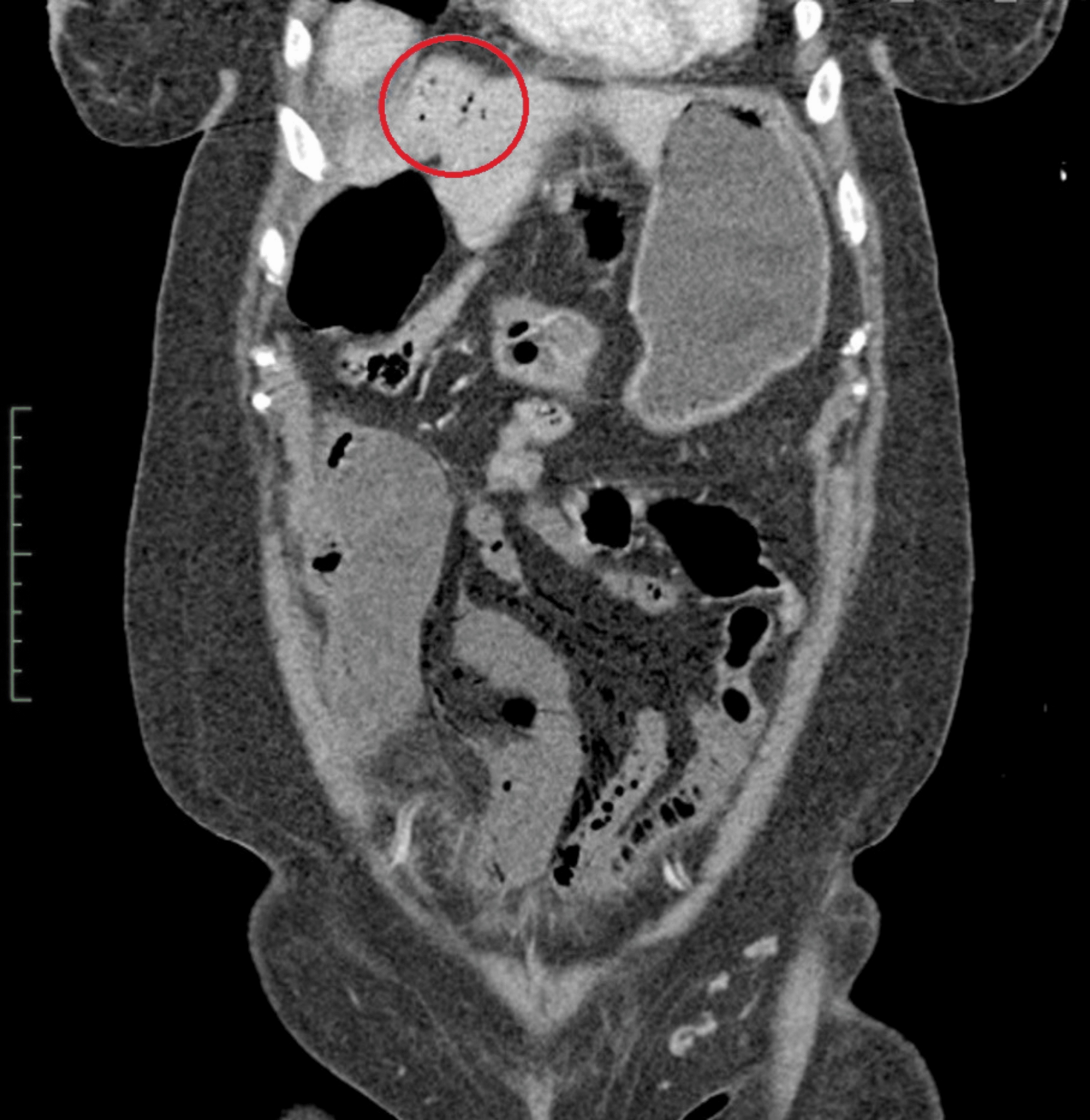
Coronal CT scan view of the abdomen and pelvis illustrating portal venous gas (red circle)

A CT scan of the abdomen and pelvis with IV contrast revealed nonenhancing, fluid-filled loops of small bowel with surrounding fat stranding (Figure 1) and subtle pneumatosis intestinalis (Figure 2). These findings were consistent with ischemic bowel, possibly complicated by necrosis. The scan also showed extensive mesenteric intravascular gas (Figure 3) and portal venous gas (Figure 4).

**Figure 5:**
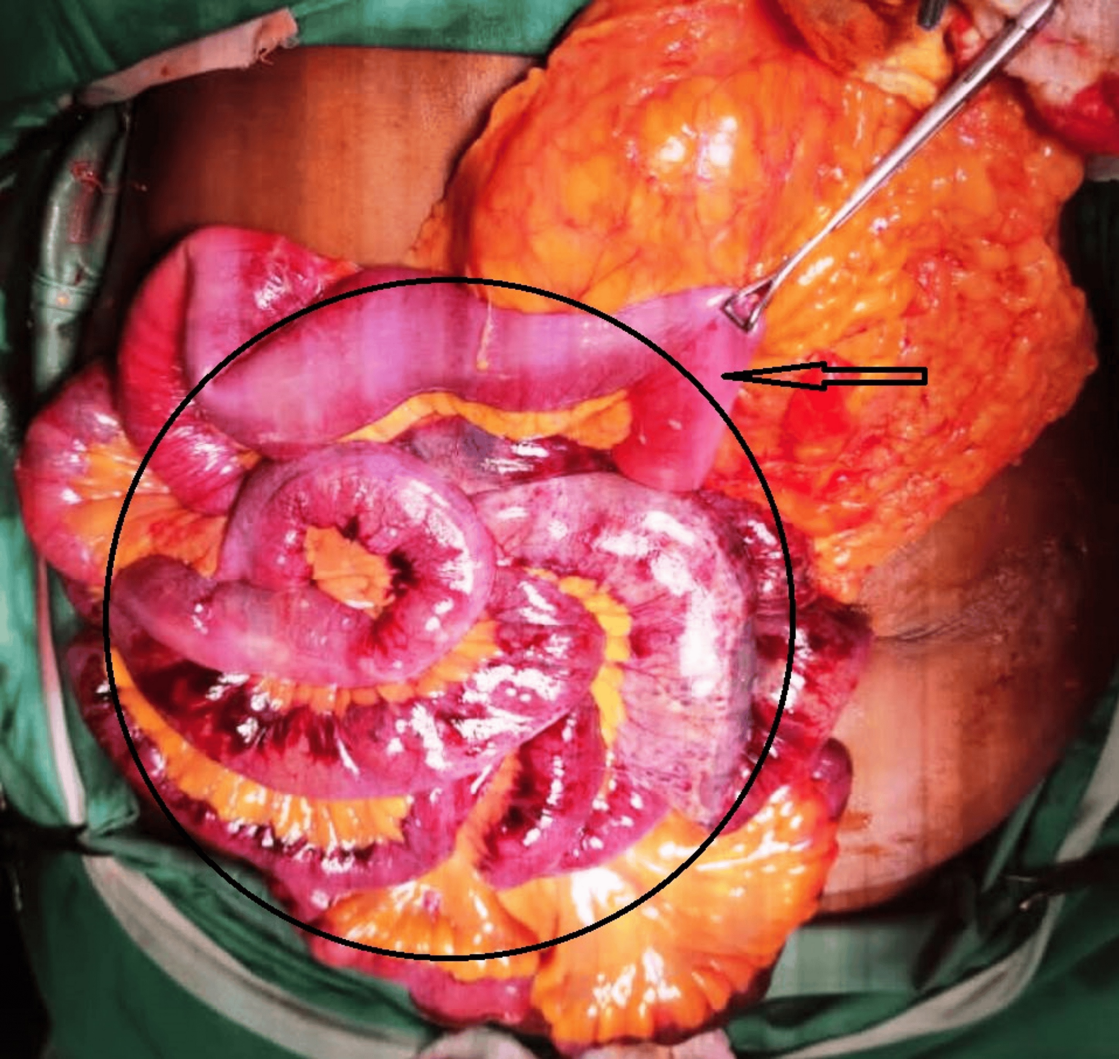
Intraoperative photograph showing the distribution of necrotic small bowel (black circle) and the ischemic starting point located 15 cm from the duodenojejunal flexure (black arrow)

**Figure 6:**
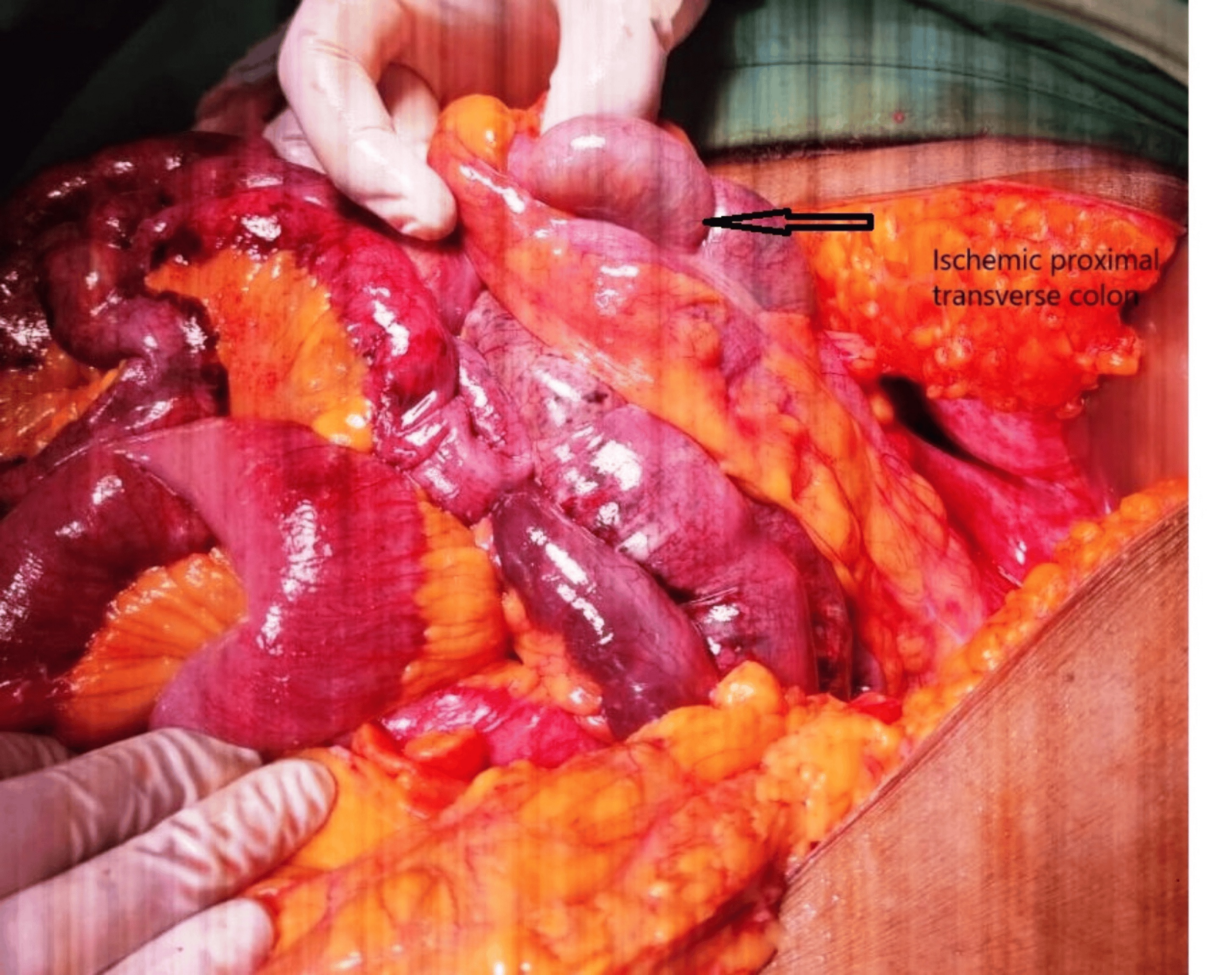
Intraoperative photograph depicting ischemic colon extending up to the proximal transverse colon (black arrow)

**Figure 7:**
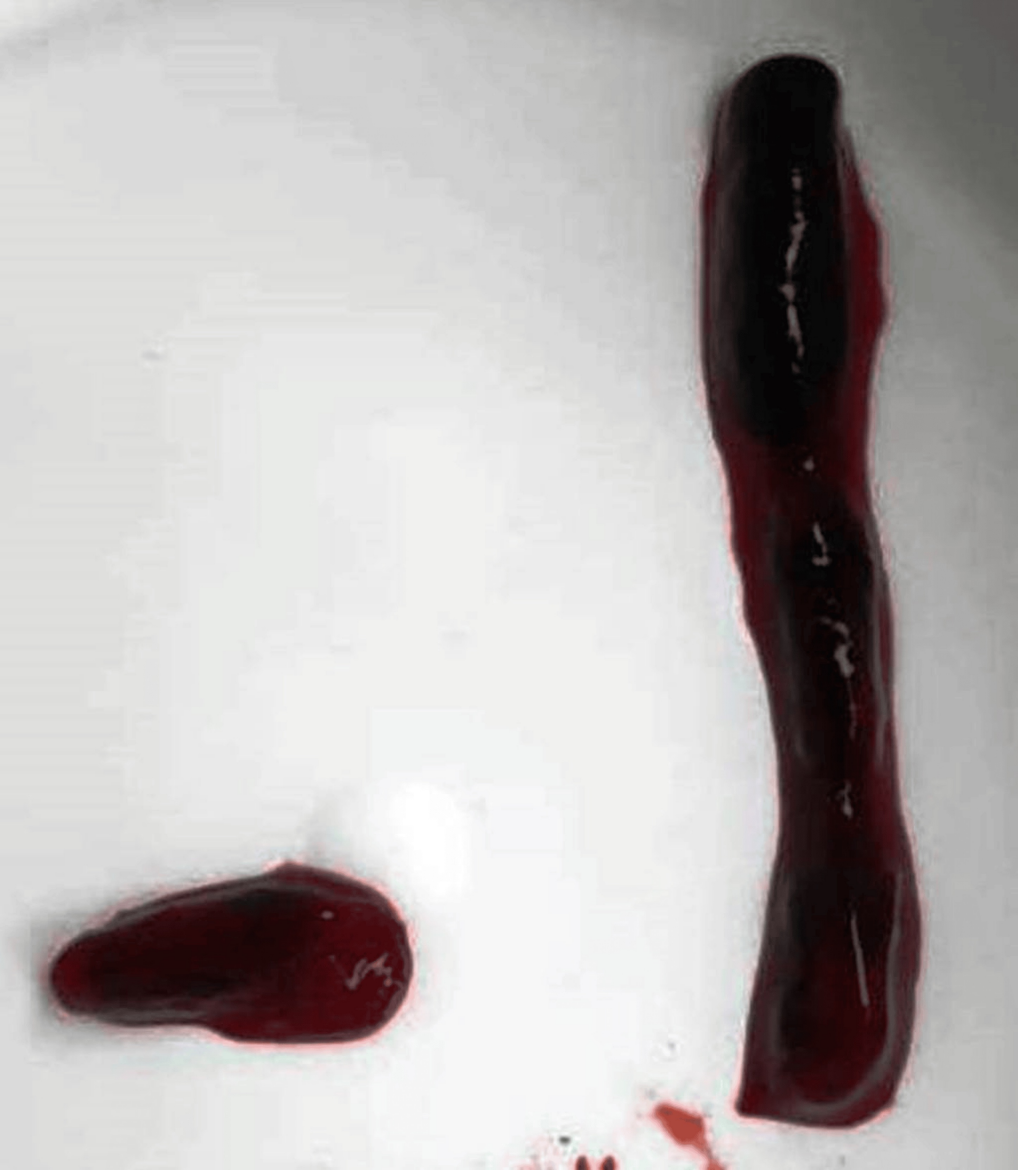
Anterior view of the surgically removed blood clots (thrombi) from the superior mesenteric artery

The patient and her family were thoroughly counseled in the presence of the anesthesiologist, and high-risk consent was obtained for exploratory laparotomy. Intraoperative findings revealed approximately 300 mL of bloody fluid in the abdomen and ischemic, devitalized small bowel (jejunum and ileum) starting 15 cm from the duodenojejunal flexure (Figure 5), along with ischemic cecum, ascending colon, and proximal transverse colon (Figure 6). Further exploration identified thrombosis of the superior mesenteric artery (SMA). Resection of the affected small and large bowel, embolectomy of the SMA (Figure 7), and Bogota bag closure of the abdomen were performed as part of damage control laparotomy.

Intraoperatively, the patient was on inotropic support and was admitted to the ICU. A therapeutic dose of heparin infusion was started during surgery and continued to maintain an activated partial thromboplastin time of 70-80 seconds. Broad-spectrum antibiotics were also administered. On postoperative day two, inotropic support was weaned off, and the patient was taken back to the operating room for a second-look laparotomy. No further ischemic segments of the small or large bowel were observed, so a primary jejunum-to-transverse colon anastomosis was performed, and the abdomen was closed with mass closure. The patient was then readmitted to the ICU for continued postoperative care. TPN was initiated, and the patient was referred to the dietitian.

The patient spent a total of eight days in the ICU before being transferred to the surgical ward. Care continued with resuscitation and multidisciplinary management involving surgical, medical, cardiac, nutrition, and dietetics teams. TPN was maintained for 32 days with gradual weaning, while nasogastric feeds were introduced on postoperative day four and oral fluids by day fifteen. Oral intake was progressively advanced to full solids, with improved absorption reflected by a change in stool pattern from multiple episodes of watery stool to soft, formed stools by day 22 post-admission and surgery.

The patient was successfully discharged to home care with outpatient follow-up on day 32 post-admission. More than two years later, she remains well and requires minimal assistance, only taking regular multivitamins.

## Discussion

SBS is classified based on anatomical, pathophysiological, and postoperative factors. Anatomically, SBS is divided into three types: Type 1: end jejunostomy without colon; Type 2: jejunocolic anastomosis without an ileo-cecal valve; and Type 3: jejunoileal anastomosis with an intact colon [6].

Pathophysiologically, SBS can be grouped by the presence or absence of the colon. The main mechanism leading to chronic intestinal failure (CIF) in SBS is malabsorption, caused by a reduction in the intestinal absorptive surface and accelerated intestinal transit. Effective rehabilitation depends on managing the three phases of SBS: the acute phase (lasting three to four weeks), the adaptation phase (one to two years), and the maintenance phase, which involves specialized diets, oral or intramuscular nutrient supplementation, and pharmacological treatments [6]. Patients who fail to adapt enter CIF.

Intestinal failure secondary to SBS is a severely disabling condition that impairs social integration and quality of life. Although not always fatal, it carries risks of serious, life-threatening complications. The main contributors to the high mid-term mortality are bacterial overgrowth and catheter-related sepsis [2].

The European Society for Clinical Nutrition and Metabolism guidelines classify intestinal failure into three types: Type I is an acute, short-term, usually self-limiting condition; Type II is a prolonged acute condition affecting metabolically unstable patients requiring complex multidisciplinary care and IV supplementation for weeks or months; and Type III is CIF in metabolically stable patients, needing IV supplementation over months or years, which may be reversible or irreversible [4].

Most adult causes of SBS include surgical resection, Crohn’s disease, trauma, malignancy, radiation, mesenteric ischemia, and iatrogenic postoperative vascular or obstructive complications [1,5]. Globally, precise incidence data on SBS remain unavailable, especially when including patients on home parenteral nutrition, those with CIF dependent on parenteral nutrition, and those weaned off parenteral nutrition [7]. Some suggest the number of SBS patients may be 1.4 times greater than those with SBS-associated CIF [8].

A key prognostic factor for long-term parenteral nutrition in SBS is the length of residual small bowel, particularly if less than 50% remains continuous with the colon. Survival rates for SBS patients dependent on parenteral nutrition have been reported as 80% at two years and 70% at five years. With adequate management, 50-70% of patients on TPN can be successfully weaned, with children generally showing better outcomes [4].

Patients facing the greatest nutritional challenges typically have one of the following surgical outcomes: duodenostomy or jejunoileal anastomosis with less than 35 cm of residual small intestine; jejunocolic or ileocolic anastomosis with less than 60 cm; or end jejunostomy with less than 115 cm of residual small intestine [8].

Studies reveal that only 50% of SBS patients without malignancy become nutritionally dependent, with a mean time to dependency of 19 months (range: three to 126 months). Overall survival rates at one, five, 10, and 20 years are 93%, 71%, 59%, and 28%, respectively [3].

Extreme SBS (ESBS) has additional criteria: a residual intestine shorter than 40 cm without a colon generally requires permanent parenteral nutrition or intestinal transplantation, whereas patients with over 80 cm of residual intestine with a valve and colon may achieve intestinal adaptation [4,9].

In 2018, Alastrué et al. documented three adult ESBS cases - all dependent on parenteral nutrition - who underwent surgical lengthening with the STEP procedure; one patient also had neo-valve reconstruction using ileum [9]. These patients achieved nutritional independence and appropriate anthropometric measures between the postoperative period and 72 months post-op.

Earlier literature includes a 1989 Oxford Journal report of a 32-year-old female with massive small bowel infarction, leaving 40 cm of small bowel distal to the duodenum. She was managed on TPN and successfully transitioned to enteral feeds with good nutritional status [10,11].

Hayes et al. reported a successful outcome in an 83-year-old female with massive small bowel resection secondary to an incarcerated hernia, leaving only 35 cm of small intestine but with an intact ileocecal valve and colon. She transitioned from TPN to partial parenteral nutrition twice weekly at home [12]. Our patient has only 15 cm of jejunum, no ileo-cecal valve, and a right-sided colon, well below the thresholds reported in previous studies [9-12].

Given the scarcity of data, there is limited documentation of adult ESBS cases subjected to severe surgical stress like ours yet achieving satisfactory discharge outcomes after a relatively short intestinal adaptation period with nutritional independence.

Our case demonstrates aggressive management of Type II intestinal failure despite limited guidelines, emphasizing the benefits of stringent surgical and intensive care in the immediate postoperative period. It also highlights the importance of a multidisciplinary approach, optimizing medical therapy for the underlying pathology (atrial fibrillation), nutritional support via TPN, and gradual weaning onto enteral feeding guided by the patient’s absorptive capacity, facilitating nutritional independence.

Interestingly, unlike many documented cases where intestinal failure leads to prolonged nutritional dependence lasting months to years or requires surgical lengthening, our patient did not require extended parenteral nutrition. It remains unclear from the literature if any similar ESBS cases have survived with such a minimal period of parenteral nutrition.

We present this case as proof of the value of persistence in surgical care and decision-making, even when facing increased risks of morbidity and mortality, showcasing the potential of a multidisciplinary team approach to achieve successful outcomes.

## Conclusions

ESBS is defined as having a residual intestine shorter than 40 cm without a colon, which usually necessitates permanent parenteral nutrition or intestinal transplantation. Alternatively, patients with a residual intestine longer than 80 cm with an intact valve and colon may achieve intestinal adaptation. The patient in this report met all the criteria for Type II intestinal failure and, by definition, would have required long-term parenteral nutrition. However, despite the severe intestinal pathology, she exceeded the expected clinical course and achieved adequate nutritional independence, surpassing what limited data and guidelines typically predict for such cases.
